# Decreased ferroportin in hepatocytes promotes macrophages polarize towards an M2-like phenotype and liver fibrosis

**DOI:** 10.1038/s41598-021-92839-z

**Published:** 2021-06-28

**Authors:** Chengyuan Cai, Danning Zeng, Qing Gao, Lei Ma, Bohang Zeng, Yi Zhou, He Wang

**Affiliations:** 1 Key Laboratory of Molecular Target & Clinical Pharmacology and the State Key Laboratory of Respiratory Disease, School of Pharmaceutical Sciences & The Fifth Affiliated Hospital, Guangzhou Medical University, Guangzhou, 511436 People’s Republic of China; 2 The Second Affiliated Hospital, Guangzhou Medical University, Guangzhou, 510260 Guangdong People’s Republic of China; 3 Department of Healthy Food Development, Infinitus (China) Company Ltd., Guangzhou, 510024 Guangdong People’s Republic of China; 4 Key Laboratory of Molecular Clinical Pharmacology & Fifth Affiliated Hospital, Guangzhou Medical University, Guangzhou, 511436 Guangdong People’s Republic of China

**Keywords:** Biochemistry, Cell biology, Immunology, Gastroenterology

## Abstract

Iron release from macrophages is closely regulated by the interaction of hepcidin, a peptide hormone produced by hepatocytes, with the macrophage iron exporter *ferroportin* (*FPN1*). However, the functions of *FPN1* in hepatocyte secretion and macrophage polarization remain unknown. *CD68* immunohistochemical staining and double immunofluorescence staining for *F4/80* and *Ki67* in transgenic mouse livers showed that the number of macrophages in *FPN1*^*−/*+^ and *FPN1*^*−/−*^ mouse livers was significantly increased compared to that in WT (*FPN*^+*/*+^) mice. *FPN1* downregulation in hepatic cells increased the levels of the M2 markers *CD206*, *TGF- β*, *VEGF*, *MMP-9*, *Laminin*, *Collagen*, *IL-4* and *IL-10*. Furthermore, the expression of *CD16/32* and *iNOS*, as M1 markers, exhibited the opposite trend. Meanwhile, *α-SMA* immunohistochemistry and Sirius red staining showed that the trend of liver fibrosis in *FPN1*^*−/−*^ mice was more significant than that in control mice. Similarly, in vitro* FPN1* knockdown in L02-Sh/L02-SCR liver cell lines yielded similar results. Taken together, we demonstrated that downregulated *FPN1* expression in hepatocytes can promote the proliferation and polarization of macrophages, leading to hepatic fibrosis. Above all, the *FPN1* axis might provide a potential target for hepatic fibrosis.

## Introduction

Hepatic fibrosis is an abnormal change in normal liver tissue caused by persistent necrosis, inflammation and the repair of fibrous connective tissue after chronic liver injury. As a chronic liver disease, it is characterized by hepatocyte injury and the excessive deposition of extracellular matrix. Meanwhile, liver fibrosis, as an early stage of liver cirrhosis, can also destroy normal tissue structure and cause serious damage to tissue function^1^.

Ferroportin1 (*FPN1, SLC40A1*) is the only known membrane protein that transports iron out of cells^2^. It is highly expressed in cells involved in iron uptake, storage and reuse, such as duodenal epithelial cells, hepatocytes, reticuloendothelial macrophages and placental syncytiotrophoblasts^3^. In addition to being related to iron transport, *FPN1* is closely related to inflammation. Studies have shown that inflammation can directly activate Toll-like receptors (TLRs) or induce the production of hepcidin through inflammatory factors such as *interleukin-6* (*IL-6*). Hepcidin can reduce the expression of *FPN1* through the hepcidin-*FPN1* axis. Mice die in the embryonic stage when *FPN1* is completely knocked out, but mice with specific knockout in hepatocytes can survive.

It has been reported that when one allele of the *FPN1*gene is mutated, an autosomal dominant genetic disorder called ferroportin disease (FD) occurs^4,5^. This mutation reduces iron transport, especially in reticuloendothelial macrophages, and eventually leads to iron accumulation in macrophages in the spleen, liver and bone. Due to the deficiency in *FPN1* activity in hepatocytes, iron deposition in discrete hepatocytes in liver tissue is evident even in the early stage^6^. The clinical symptoms vary, but the overall manifestation is milder than that of classical hemochromatosis (HC). Both classical forms of HFE-HC and FD are associated with antioxidant defense and organ fibrosis. However, the mechanism by which FD induces liver fibrosis remains unknown. In HC, but not in FD, a ubiquitination mutant of *FPN1* caused by an *FPN1* mutation results in a decrease in the sensitivity of the *FPN1* protein on the cell surface to hepcidin and a reduction in the iron content in macrophages.

Macrophages are important components of nonspecific immunity. Macrophages (known as Kupffer cells in the liver) play a critical role in liver fibrosis. Liver contain tissue resident macrophages that are indispensable for tissue homeostasis. It has reported that the majority of tissue-resident macrophages are initially derived from the embryonic yolk-sac and maintain via self-renewal^7^; however, this varies amongst tissues. Circulating monocytes contribute to the resident macrophage pool in some tissues, monocyte-derived macrophages (MdMs) redominantly enter tissues in states of tissue injury or inflammation^8^. Although tissue resident macrophages share functions like clearance of cellular debris and tissue remodeling, they can also exert specific tissue function^9^.

By regulating the activation or apoptosis of hepatic stellate cells and the formation and degradation of fibrocollagen, macrophages can promote or reverse the two-way regulation of liver fibrosis^10,11^. Based on their surface markers and the cytokines they secreted, macrophages can be divided into proinflammatory M1 and anti-inflammatory M2 phenotypes. Human acute monocytic leukemia cells, TPH-1, were stimulated with phorbol 12-myristate 13-acetate (PMA) to obtain macrophage-like M0 cells. M1 macrophages can induce high expression of nitric oxide synthase (*iNOS*), increase the expression of *CD16/32* on the membrane surface and promote the secretion of inflammatory cytokines, such as *IL-1β*, *IL-6*, *IL-12α*, *IL-12β*, *TNF-α* and *IFN-γ*. M2 macrophages secrete anti-inflammatory cytokines such as *IL-4*, *IL-10*, *TGF-α*, *TGF-β*, *VEGF* and *CD206*^12^. Several subtypes of M2-polarized macrophages (M2a, M2b, M2c) can be further discriminated depending on their phenotype and functional properties. M2a macrophages are activated by IL-4/IL-13 and possess tissue repair and immunoregulating features. M2b macrophages are stimulated by immune complexes and support humoral immunity and allergic reactions. Stimulation by IL-10 or glucocorticoids favors the activation of M2c phenotype, which induces anti-inflammatory reactions by remodeling of extracellular matrix and suppression of immunity^13^. In the acute stage of inflammation, M2 macrophages can reduce the secretion of proinflammatory cytokines and alleviate inflammation damage, but in the chronic stage of inflammation, M2 macrophages can secrete profibrotic factors, including typical *TGF-β*, which can promote the activation of myofibroblasts and the synthesis of extracellular matrix and promote the development of fibrosis.

The purpose of this study was to investigate the relationship between the expression of *FPN1* in hepatocytes and hepatic fibrosis and the role of these hepatocytes in macrophage proliferation and polarization and to explore which factors are involved in these processes.

## Results

### FPN1 regulated macrophage proliferation and polarization

**Figure 1:**
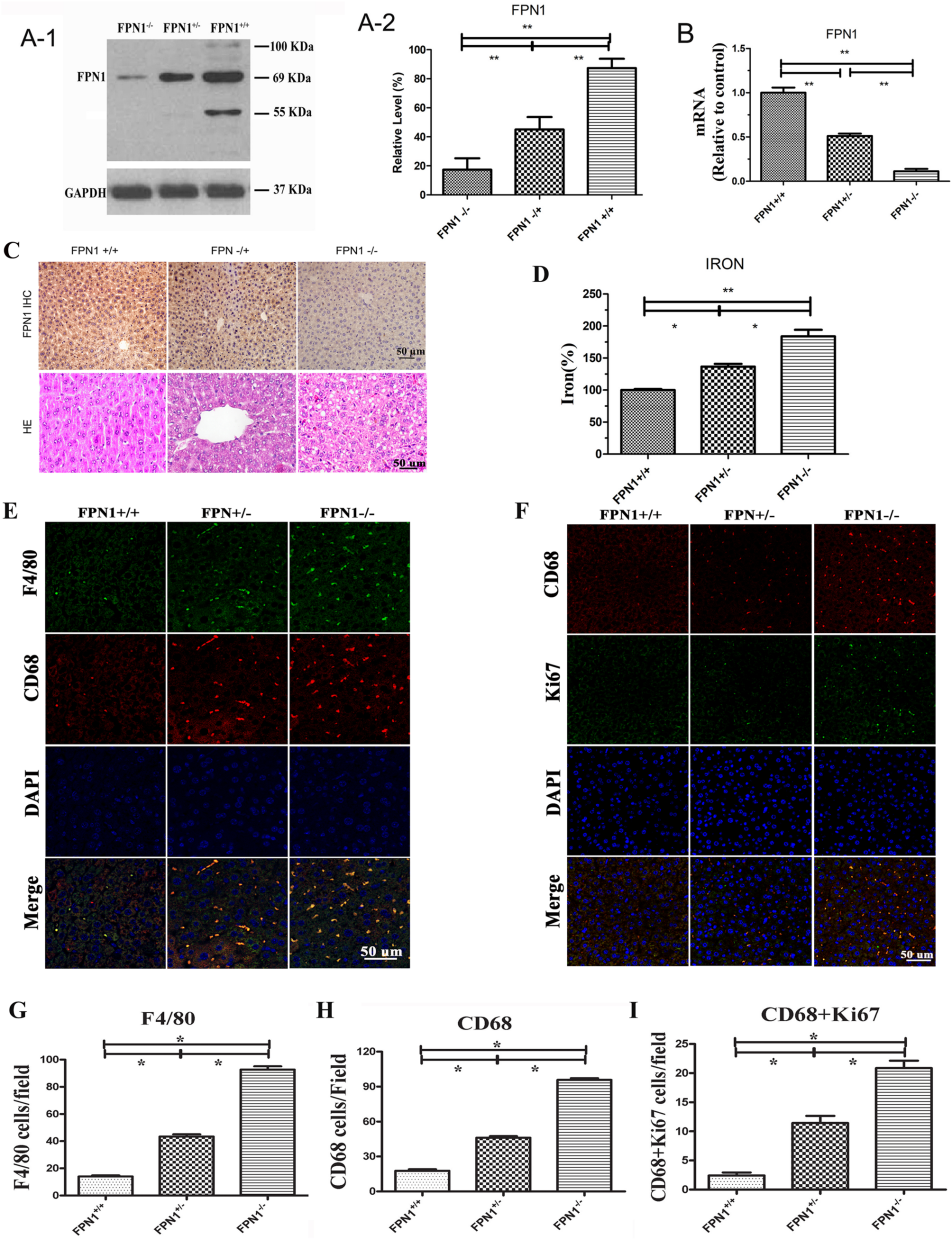
Downregulation of *FPN1* in hepatic cells induced macrophage proliferation in vivo. Western blot (**A**), Q-PCR (**B**), HE staining and immunohistochemical (**C**) analysis of the expression of FPN1 in hepatocytes of transgenic mice. (D) The iron content in mouse livers (*FPN1*^+/+^, *FPN1*^−/+^, and *FPN1*^−/−^) using the iron assay protocol based on ferrous iron (Fe2+) reacting with Ferene S to produce a stable colored complex with absorbance at 593 nm. (**E**) F4/80 (green) and CD68 (red) staining of liver sections. Total nuclei were costained with DAPI (blue). (**F**) Ki67 (green) and F4/80 (red) staining of liver sections. Total nuclei were costained with DAPI (blue) (× 400). Quantification analysis of F4/80-positive (**G**), and CD68-positive (**H**) nuclei and F4/80 + Ki67-positive (**I**). All results are from three independent experiments. *P < 0.05, **P < 0.001.

**Figure 2:**
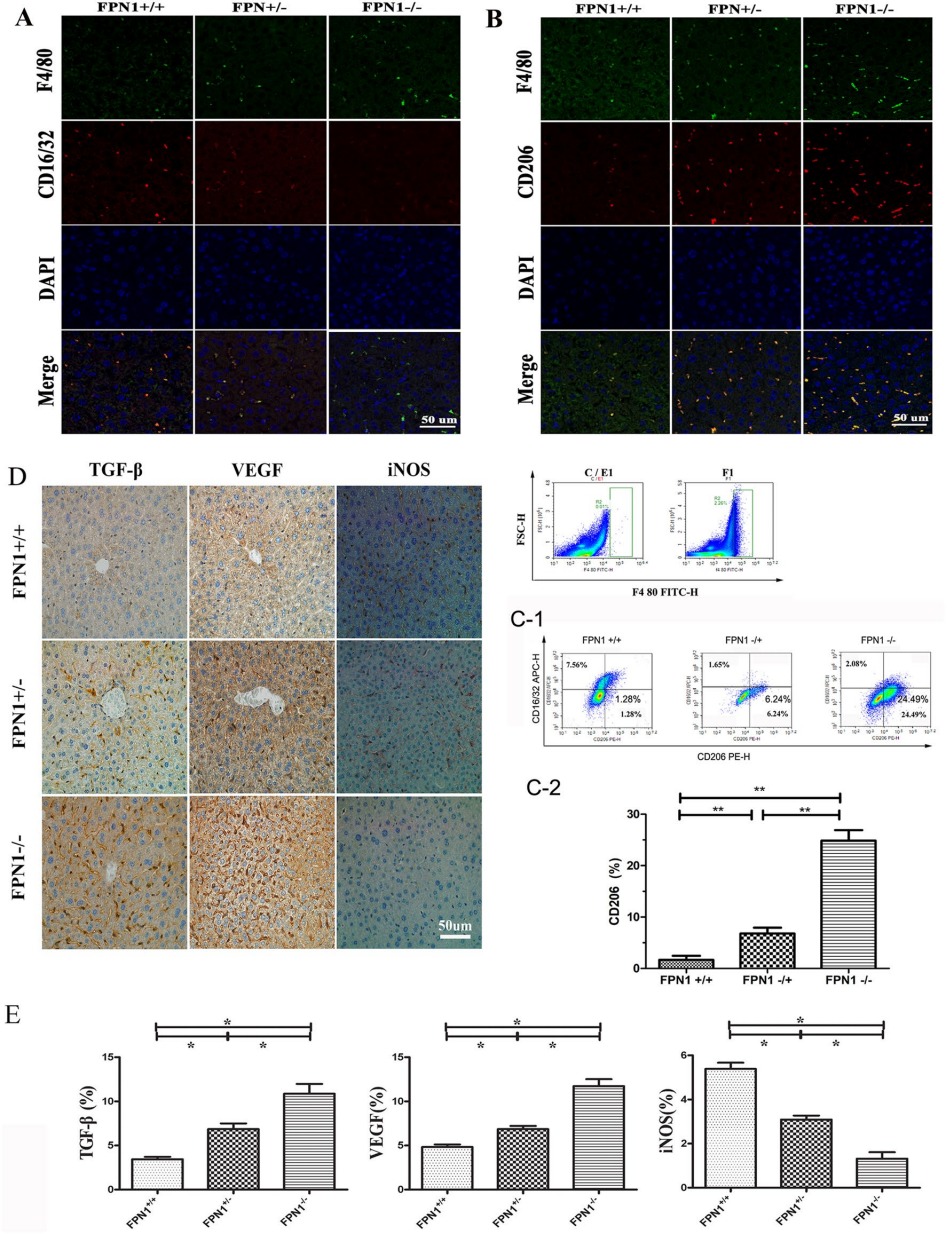
Downregulation of *FPN1* in liver cells promoted M2 macrophage polarization in vivo. Liver sections were co-stained with F4/80 (**A**) and CD206 or CD16/32 (**B**). M1 macrophages were F4/80^high^ CD16/32^high^, and M2 macrophages were F4/80^high^ CD206^high^. (**C**) FCM analysis of the expression of specific biomarkers of macrophages in mouse livers. (1) Image of FCM. Single macrophage first identified by forward scatter height (FSC-H) versus F4/80^+^ gating using in the IgG control and F4/80 stained mouse liver cells; (2) CD206 expression level of FCM. (**D**) IHC analysis of the expression of M1 and M2 marker proteins in mouse livers. (1) Image of IHC; (2) TGF-β, VEGF and iNOS level of IHC. All results are from three independent experiments. *P < 0.05, **P < 0.001.

Liver-specific FPN1 knockout mice were made by crossing Albumin-Cre mice with *FPN1*^*flox/flox*^ mice generating wild-type (*FPN1*^+*/*+^), heterozygous (*FPN1*^*−/*+^), and homozygous knockout (*FPN1*^*−/−*^) mice used in this study (Fig. 1A–C). It has reported that the predicted molecular mass of purified human FPN1 is approximately 69-kDa. Yet, in mouse tissues, different FPN1 molecular weight (MW) forms have been detected^14^. As *FPN1* expression was downregulated in liver cells, the iron content in liver cells was increased (Fig. 1D). To test whether silencing *FPN1* in hepatic cells contributed to the changes in macrophages, double immunofluorescence staining for *F4/80* and *CD68* immunohistochemical staining and double immunofluorescence staining for *CD68* and *Ki67* were performed in liver sections from WT (*FPN*^+*/*+^) and *FPN1* knockout mice (Fig. 1E,F). The results showed that the number of macrophages in *FPN1*^*−/*+^ and *FPN1*^*−/−*^ mouse livers was significantly increased compared to that in *FPN*^+*/*+^ mouse livers (Fig. 1G–I). Macrophage polarization by was detected by immunofluorescence staining and flow cytometry in liver tissues from *FPN*^+*/*+^*, FPN1*^*−/*+^ and *FPN1*^*−/−*^ mice. It was shown that macrophages in *FPN1*^*−/−*^ and *FPN1*^*−/*+^ mice were obviously polarized to the M2 subtype (Fig. 2A–C). Among all livers, the percentage CD206 in *FPN1*^*−/−*^ mice was 3.94-fold and 19.1-fold higher than that in *FPN1*^*−/*+^ and *FPN*^+*/*+^ mice, respectively (Fig. 2C). We also found that as *FPN1* expression decreased in mouse livers, M2 marker proteins such as *TGF-β* and *VEGF* were increased significantly, while the expression of *iNOS*, as a M1 marker protein, exhibited the opposite trend (Fig. 2D). The above results indicated that the knockdown of *FPN1* in liver cells induced macrophage proliferation and M2 polarization.

### FPN1 downregulation in hepatic cells induced hepatic fibrosis

**Figure 3:**
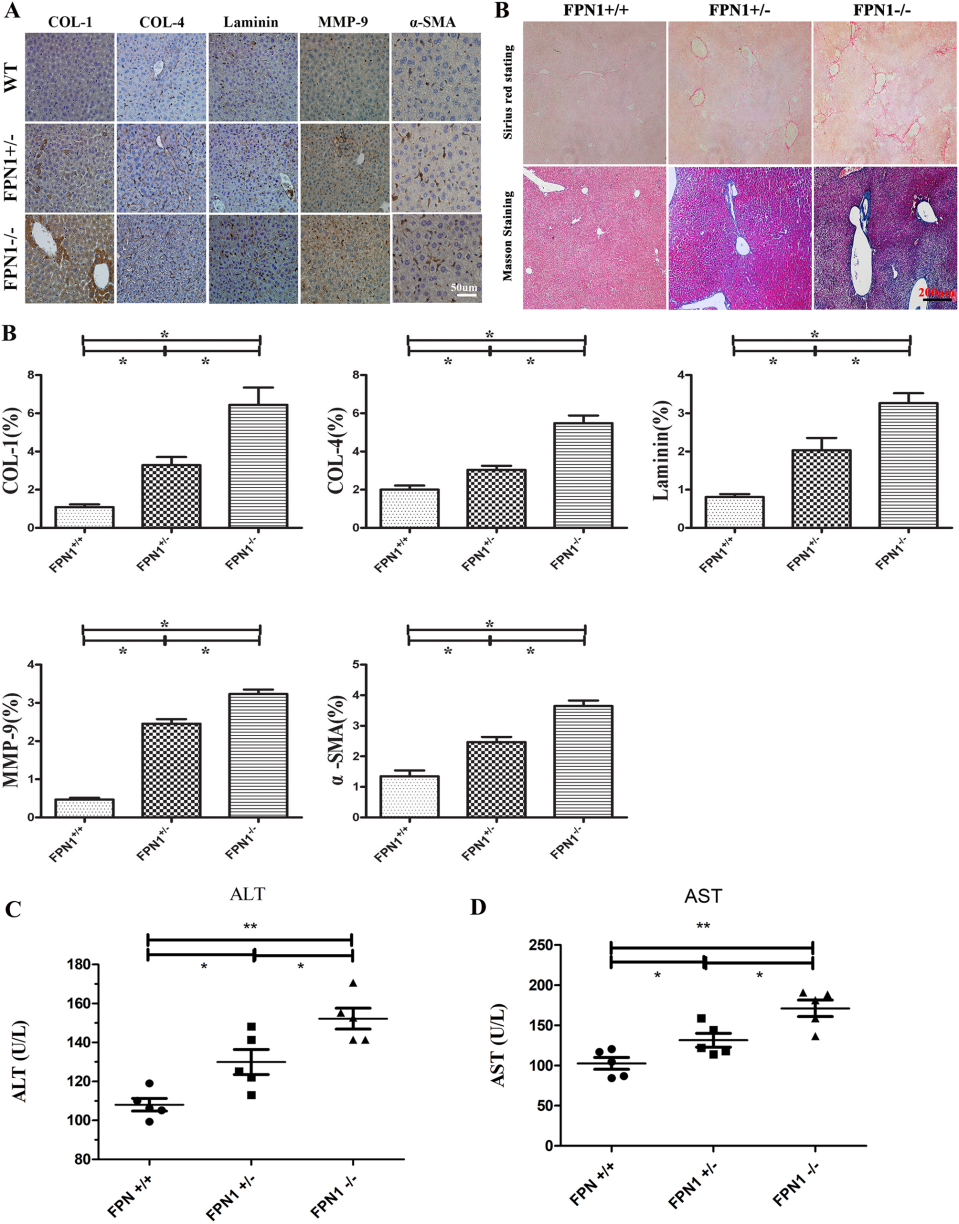
Downregulation of *FPN1* in hepatic cells induced liver fibrosis. (**A**) Liver sections were incubated with antibodies against COL-1, COL-4, Laminin, α-SMA, and MMP-9 and stained with Sirius red or Masson (× 400). (**B**) Error bars correspond to the mean ± standard deviation of IHC. ALT (**C**) and AST (**D**) content in mouse serum (*FPN1*^+/+^, *FPN1*^−/+^, *FPN1*^−/−^). All results are from three independent experiments. *P < 0.05, **P < 0.001.

Macrophage polarization is closely related to fibrosis^15^. Immunohistochemical analysis showed that compared to that in the *FPN*^+*/*+^ group, the expression of *Collagen 1, Collagen 4* and matrix metalloproteinase (*MMP-9*) in *FPN1*^*−/−*^ and *FPN1*^*−/*+^ mouse livers were enhanced, indicating that the extracellular matrix of liver tissues increased. Furthermore, additional markers of fibrosis were tested. As expected, the levels of *Laminin* and *α-SMA* were increased. Extracellular fibrotic deposition, detected by Sirius red staining and Masson staining, further confirmed that the inhibition of FPN1 impaired hepatic fibrosis in vivo (Fig. 3A,B).

Glutamic-pyruvic transaminase (*ALT*) and glutamic oxalacetic transaminase (*AST*) were regards as important index for liver fibrosis or injury. A lots of *ALT* or *AST* are released into the blood and induced increased expression of *ALT* or *AST* in serum when there are hepatocyte necrosis, liver fibrosis or injury. As is shown in Fig. 3C,D further confirm that *FPN1* downregulation in hepatic cells induced hepatic fibrosis.

### FPN1 regulated macrophage polarization to the M2 phenotype by inducing IL-10 and TGF-β expression in vivo

**Figure 4:**
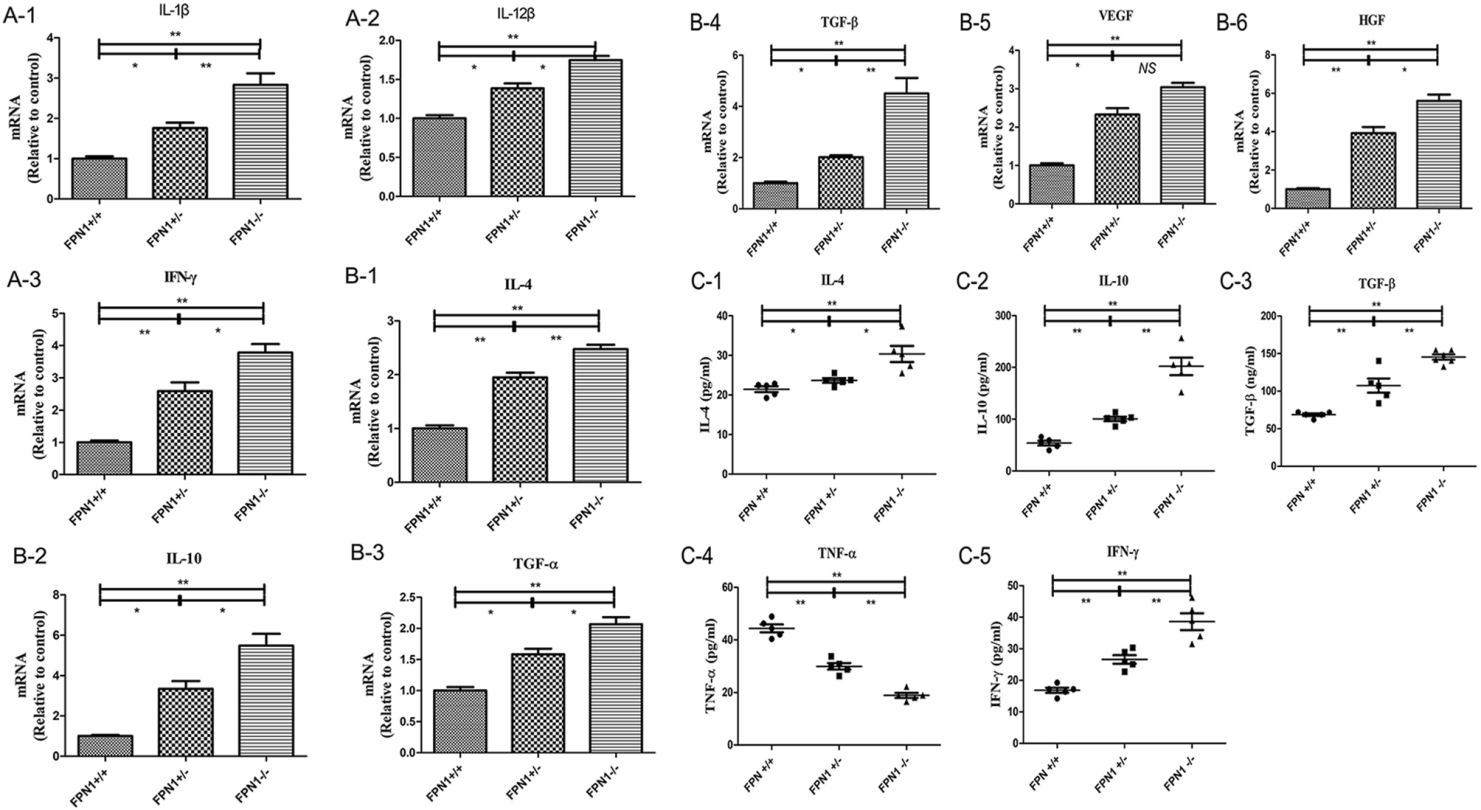
*FPN1* downregulation in hepatic cells increased the expression of specific biomarkers of M2 macrophages in vivo. (**A**,**B**) Q-PCR analysis of the expression of specific biomarkers in mouse livers. (**C**) ELISA analysis of the expression of specific biomarkers in mouse serum. All results are from three independent experiments. *P < 0.05, **P < 0.001.

As mentioned above, we found that the downregulation of *FPN1* expression in mouse liver cells promoted macrophage M2 polarization and fibrosis. To investigate which cytokines were involved in this process, Q-PCR and ELISA were used. The Q-PCR results demonstrated that compared to that in *FPN*^+*/*+^ or *FPN1*^*−/*+^ mice, the expression of *IL-1β, IL-4, IL-10, IL-12β, TGF-α, TGF-β, HGF, VEGF,* and *IFN-γ* was increased in *FPN1*^*−/−*^ hepatocytes (Fig. 4A,B). Though *IL-6, IL-12α, TNF-α* and *CSF-1* showed no significant differences in *FPN1*^*−/−*^ and *FPN1*^*−/*+^ hepatocytes (Supplementary Fig. S1–4), the expression of *IL-10 or TGF-β* in *FPN1*^*−/−*^ hepatocytes showed 5.5-fold or 4.2-fold higher than that in *FPN*^+*/*+^ hepatocytes. Their ratio was higher than those of *IFN-γ* and *IL-12β* in *FPN1*^*−/−*^ hepatocytes *again,* which were 3.5-fold and 1.6-fold higher than that in *FPN*^+*/*+^ hepatocytes, suggesting that FPN1 significantly regulated macrophage polarization to the M2 phenotype, compared with M1 phenotype. Moreover, the ELISA results indicated that the levels of *IL-10*, *TGF-β,* and *IL-4* were significantly increased in *FPN1*^*−/−*^ and *FPN1*^*−/*+^ mice (Fig. 4C). These cytokines are strong stimulators of M2 polarization and tissue remodeling and repair^16^. These data suggested that *FPN1* induced macrophage polarization and fibrosis through *IL-10* and *TGF-β*.

### FPN1 was involved in macrophage polarization in vitro

**Figure 5:**
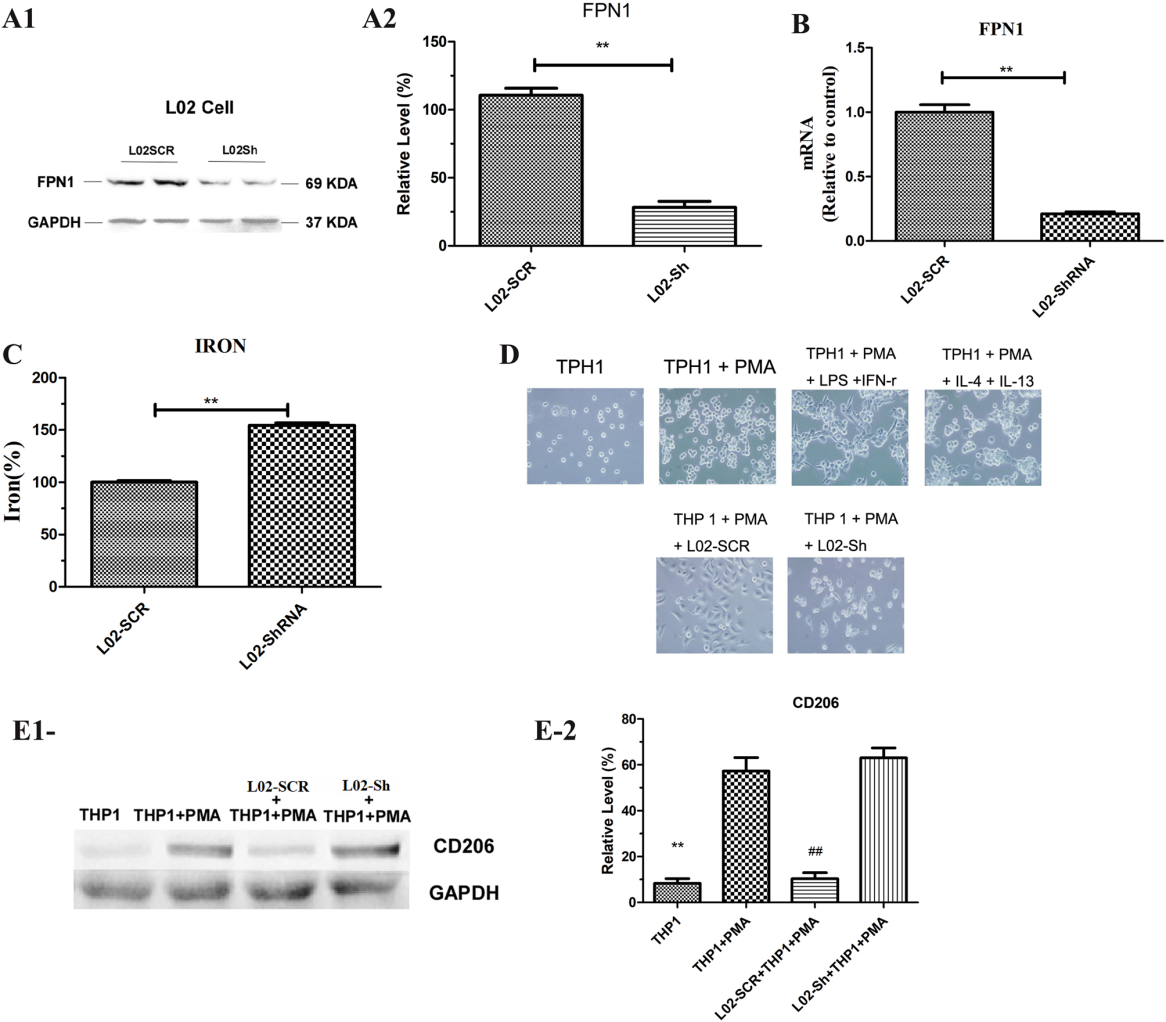
Downregulation of *FPN1* in L02 cells promoted M2 macrophage polarization in vitro. Western blot (**A**) and Q-PCR (**B**) analysis of the expression of *FPN1* in L02-SCR and L02-Sh cells. (**C**) Iron content in L02-SCR and L02-Sh cells. (**D**) THP-1 cells plated in culture plates were incubated with 160 nM PMA in RPMI 1640 medium containing 5% FBS for 48 h. For M1 macrophage induction, 10 pg/ml LPS and 20 ng/ml *IFN-γ* were added to the medium without PMA. For M2 macrophage induction, 20 ng/ml *IL-4* and 20 ng/ml *IL-13* were added to the medium. (**E**) Western blot analysis of *CD68*, *CD11b*, *CD206* and *iNOS* expression in macrophages. (1) Image of western blot; (2) Grey level of western blot. All results are from three independent experiments. **P < 0.001. **P < 0.001, vs THP1 + PMA. ^##^*P* < 0.001, vs L02-Sh + THP1 + PMA.

In order to further confirm the role of FPN1 in macrophage polarization, L02 cells were used in vitro to further study FPN1 knockdown using short-hairpin (Sh) or scrambled (Scr) RNA to interfere with endogenous FPN1 (Fig. 5A,B), this was supported by increase of iron in L02-Sh (Fig. 5C). Interestingly, compared with TPH1 + PMA + L02-SCR group, it is showed for the M2 polarization in TPH1 + PMA + L02-Sh group and TPH1 + PMA + IL-4 + IL-13 group (Fig. 5D), indicated decreased FPN1 promoted liver cancer cell M2 polarization. PMA treatment enhanced the adherence of the THP1 cells. Two morphological characteristics of THP-1 cells following differentiation is an increase in cytoplasmic volume and granularity (Fig. 5D)^17^. M0 macrophages were cocultured with L02-SCR and L02-Sh cells. We detected M2 macrophages with a *CD206* antibody. The in vitro results were consistent with the in vivo results. The expression of *CD206* was significantly increased in M0 macrophages cocultured with L02-Sh cells (Fig. 5E), suggesting that macrophages exhibited M2 polarization.

**Figure 6:**
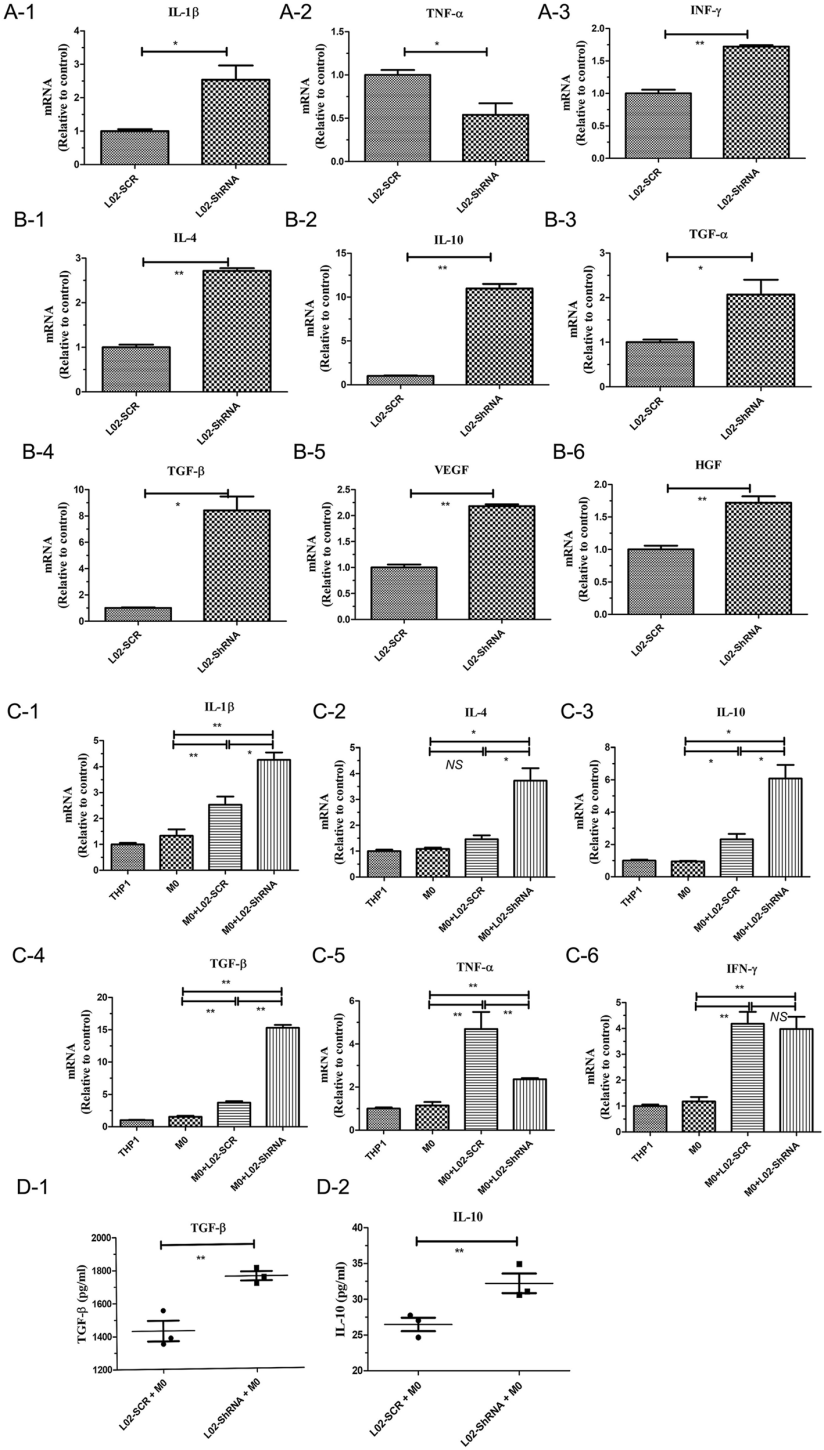
Silencing *FPN1* increased the expression of specific biomarkers of M2 macrophages in vitro. (**A**–**C**) Q-PCR analysis of the expression of specific biomarkers in L02-Sh cells, L02-SCR cells + M0 macrophages, and L02-Sh cells + M0 and macrophages. (**D**) ELISA analysis of the expression of specific biomarkers in the culture medium. All results are from three independent experiments. *P < 0.05, **P < 0.001.

Many reports have indicated that the expression of *TNF-α* and *IL-6* are increased in M1 macrophages^18^ and that *IL-10* and *TGF-β* expression is induced by M2 polarization^19,20^. Thus, we detected the expression levels of these genes in different liver cells and macrophages. The Q-PCR results showed that many cytokines, such as *IL-4*, *IL-10*, *TGF-α, TGF-β and VEGF*, were successfully induced in L02-Sh cells compared with L02-SCR cells (Fig. 6A,B; Supplementary Fig. S5–8). Interestingly, when L02-Sh cells were cocultured with M0 macrophages, the increasing expression of *IL-4, IL-10 and TGF-β* were significantly showed in L02-Sh + M0, compared with L02-SCR + M0, on the contrary, these results were not showed in *TNF-α* and *IFN-γ*, indicated that the high expression of cytokines in the L02-Sh cells promoted M0 to M2 polarization (Fig. 6C), and this was further confirmed by ELISA analysis (Fig. 6D; Supplementary Fig. S9–11). This indicated decreased *FPN1*-induced M2 macrophage polarization.

## Discussion

Macrophages are among the most versatile cells in the body^21^. Heterogeneity arises due to macrophage differentiation from monocyte precursors, and the phenotype of macrophages is determined by genetic modification as well as specific tissue-related and immune-related stimuli. Macrophages can be labeled by *CD68* or *F4/80*. M1 macrophages are usually characterized by high expression of CD16/32 and proinflammatory cytokines, including *IL-1β, IL-12* and *TNF-α*. M2 macrophages exhibit high expression of CD206 (mannose receptor) and anti-inflammatory cytokines (*IL-4, IL-10, TGF-β,* and *VEGF*). M2 macrophages can secrete profibrotic factors that can promote the activation of myofibroblasts, the synthesis of extracellular matrix, and the development of fibrosis^22^.

The involvement of *FPN1* in immune regulation is complex. In recent years, studies have shown that *FPN1* is an anti-oncogene in breast cancer and myeloma, but not in the liver^23,24^. Based on our current findings, the downregulation of *FPN1* in liver cells promotes M2 macrophage proliferation and polarization in vivo***.*** Moreover, the expression of M2 markers (*CD206, TGF-β,* and *VEGF*) increased significantly, suggesting that macrophages in the livers of mice had an obvious tendency to polarize towards the M2 phenotype; meanwhile, the expression of *CD16/32* and *iNOS*, as M1 marker proteins, exhibited the opposite trend. Additionally, macrophages were labeled with *CD68* and *F4/80*, and the results showed that the number of intrahepatic cells and macrophages increased significantly as *FPN1* expression decreased in mouse hepatocytes.

The two main contributors to hepatic fibrosis are excessive fibroblast proliferation and matrix accumulation^22^. The onset of these processes is usually preceded by an acute inflammatory response. Alpha-smooth muscle actin (*α-SMA*) is a marker of the activation of hepatic stellate cells^16^. Additionally, the activation of hepatic stellate cells can promote transformation into muscle fibrosis cells and ultimately promote the formation of liver fibrosis. *TGF-β* is a known mediator of fibrotic remodeling and matrix accumulation, and the secretion of *TGF-β* by fibroblasts can directly promote collagen synthesis and maturation^25^. This is consistent with the increase in collagen in *FPN1* knockout mice. Vascular endothelial growth factor (*VEGF*) can promote the proliferation of vascular endothelial cells and is also the strongest angiogenic factor. The increased expression of vascular endothelial growth factor in the livers of transgenic mice may be due to hypoxia in local pathological tissues, which stimulates the activation of hepatic stellate cells and then produces a variety of cytokines, resulting in increased expression of vascular endothelial growth factor. Additionally, *VEGF* can induce endothelial cells to express plasminogen activator and matrix collagenase and then promote peripheral vascular growth^16^. The significant correlation between the levels of *VEGF* and *TGF-β* in mouse livers is mainly due to the direct activation of vascular endothelial growth factor when the expression of *TGF-β* increases in vivo^26^. Hepatocyte growth factor (*HGF*) is a multifunctional antifibrotic factor involved in kidney development, acute injury and regeneration^27,28^. Increased expression of *HGF* and *MMP-9* can inhibit *ECM* production^29^. *FPN1* downregulation in hepatic cells increases the expression of *Collagen*, *Laminin, α-SMA, TGF-β* and *VEGF*, and induces a state of immune tolerance in the livers of mice^30^. Handa P 's groups have shown that dietary iron overload of C57Bl/6 mice led to hepatic macrophage M1 activation and stimulated hepatic fibrogenesis^31^. However, there is a dynamic balance between M1 and M2 macrophages in the process of fibrosis. The main differences between dietary iron overload model and our hepatocyte specific FPN1 knockout mice model was iron content in macrophages depending on iron homeostasis in liver microenvironment, resulting in entirely different polarization.

*IL-4*, *IL-10* and *TGF-β* can inhibit the expression of *TNF-α*, *IL-6* and *IL-12*, leading to the development of a liver microenvironment that favors the M2 polarization of macrophages^32,33^. *IL-1β* can attenuate collagen deposition mediated by *TGF-β*, which indicates that it has a long-term antifibrotic effect on some tissues^34^. The increased expression of *IL-1β* may attenuate tissue remodeling mediated by *TGF-β* in the absence of *FPN1*. Though decreased *FPN1* also induce the increase of *IFN-γ* and *IL-12β*, their magnitude of increase was lower than that in *IL-10* and *TGF-β*. This indicated that M2 phenotype macrophages were mainly showed in *FPN1*^*−/−*^ mice. In addition, no significant different *TNF-α* and *IFN-γ* protein expression between L02-Sh and L02-SCR cells is not accordance with significant difference between *FPN1*^*−/−*^ mice and *FPN1*^+*/*+^ mice, suggested that the protein *of TNF-α* and *IFN-γ* can easily degraded in vitro. Together*,* the elevated levels of *IL-4*, *IL-10* and *TGF-β* observed in our *FPN1*^*−/−*^ animal models may be a response to an increase in M2-mediated remodeling processes that occur in the absence of *FPN1*.

THP-1 can usually be induced by PMA to obtain macrophages^35^. Furthermore, *IL-4* + *IL-13* would transfer M0 to M2 (Supplementary Fig. S12). Interestingly, decreased FPN1 in L02 cells promoted M2 polarization of THP-1 cells as measured by increased CD206, indicating IL-4 and IL-13 cytokines play a key role again.

Previous studies on *FPN1* were mainly related to iron^24^. In this study, we found that *FPN1* is also closely related to macrophage proliferation, polarization and fibrosis. Reduced expression of *FPN1* in hepatocytes promotes iron accumulation in hepatocytes. This result is not contradiction with previous studies, which have shown that iron-overloaded macrophages exhibit an M1 phenotype^36^, because elevated iron was showed in hepatocytes, or not macrophage in *FPN1*^*−/−*^ mice. Whether it acts macrophage would be further studied. Whether the decrease in *FPN1* expression further leads to hepatocellular carcinoma after the formation of the M2 environment in the liver remains to be further studied. It will be very interesting to use conditional knockout mouse models to detect the physiological role of *FPN1* in hepatocellular carcinoma in the future.

**Figure 7:**
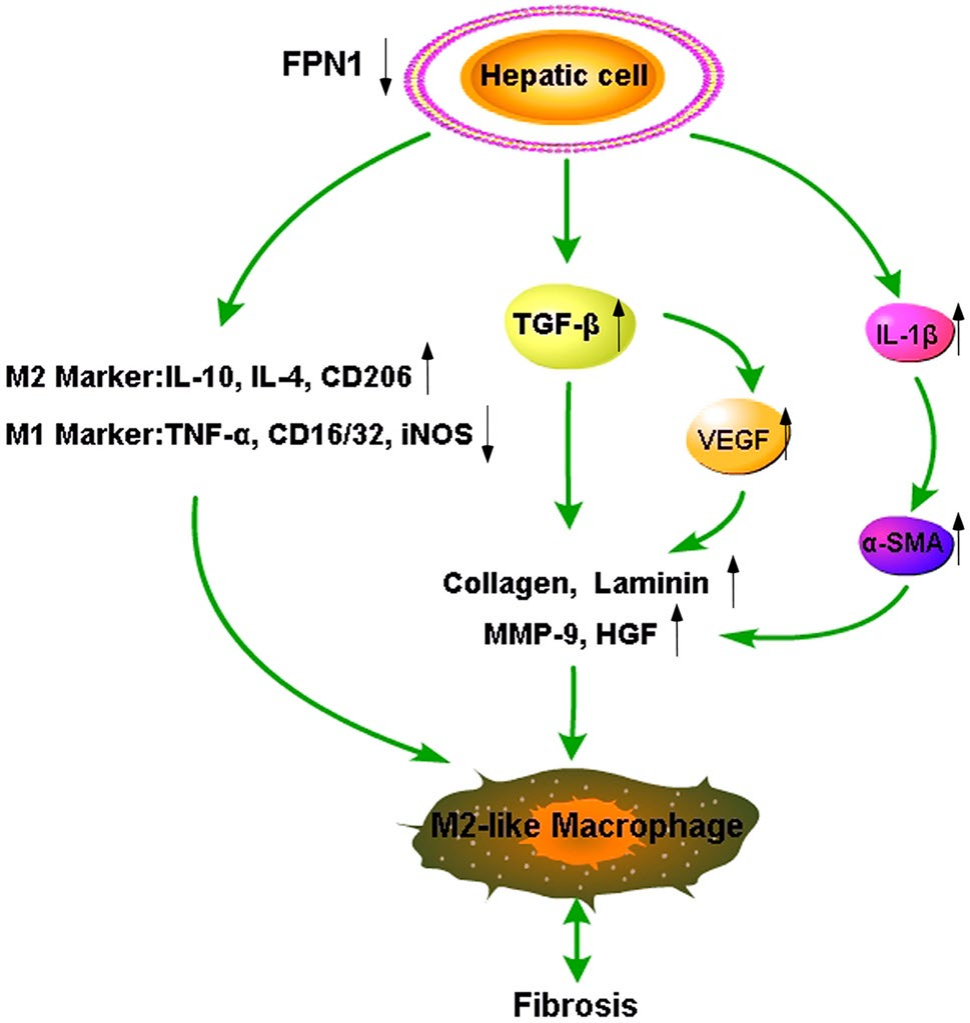
Molecular mechanism of *FPN1* involvement in macrophage polarization and hepatic fibrosis. Downregulation of *FPN1* in hepatic cells increases the levels of *IL-10* and *TGF-β*, thus inducing macrophage polarization to the M2 phenotype while promoting hepatic fibrosis.

In conclusion, the downregulation of *FPN1* in hepatocytes is beneficial to the proliferation and polarization of macrophages to the M2-like phenotype in the liver, which may lead to fibrosis (Fig. 7). Potential drugs that can upregulate the expression of *FPN1* may be new directions in the treatment of hepatic fibrosis.

## Materials and methods

### Cell culture and treatment

THP-1 (Human acute monocytic leukemia cell line) cells and L02 cells were purchased from ATCC. The cells were all cultured in RPMI 1640 containing 10% heat-inactivated FBS and 1% penicillin–streptomycin in a 5% CO_2_ atmosphere at 37 °C. THP-1 monocytes were differentiated into M0-like macrophages by incubation with 160 ng/ml phorbol 12-myristate 13-acetate (PMA, Sigma, P8139) for 48 h in RPMI medium. PMA-induced macrophages were polarized into M1 macrophages by incubation with 20 ng/ml of IFN-γ (R&D system, #285-IF) and 10 pg/ml of LPS (Sigma, #8630). Macrophage M2 polarization was obtained by incubation with 20 ng/ml of interleukin 4 (R&D system, #204-IL) and interleukin 13 (R&D system, #213-ILB). In the co-culture experiments, 0.5 × 10^6^ THP-1 monocytes were cultivated and differentiated for 48 h before being incubated respectively with 0.5 × 10^6^ L02-SCR or L02-Sh cells in 6-well Transwell plates for 48 h^37^ .

### Animals

*FPN1*^*flox/flox*^ conditional gene knockout mice were constructed by Nancy Andrews of the USA. Two LoxP sites were inserted into exon 6/7 of the *SLC40A1* gene, and the mice were maintained on a 129/SvEvTac background. *SLC40A1-LoxP* transgenic mice (129S-Slc40a1tm2Nca/J) were purchased from Jackson Laboratories (USA), backcrossed on a C57BL/6 background and bred in-house. When they were bred with liver cell-specific promoter (Alb-Cre) mice, all of the offspring obtained exhibited hepatocyte-specific knockout. Mice with downregulation of the *FPN1* gene were used as the model mice for this experiment. Unless indicated, age-matched animals were used at 8–12 weeks of age. Homozygote (*FPN1*^*−/−*^), heterozygote (*FPN1*^*−/*+^) and wild type (*FPN1*^+*/*+^) mice were obtained by mating, and the genotypes were determined by agarose gel electrophoresis. All animals in this study were raised in SPF-level animal rooms and fed a standard diet. The housing of the animals and the experiment procedures were carried out in accordance with the Guide for the Care and Use of Laboratory Animals (United States National Institutes of Health) and were approved by the Ethical Committee for Care and Use of Laboratory Animals of Guangzhou Medical University. This study was carried out in compliance with the ARRIVE guidelines 2.0.

### Real-time quantitative PCR

Total RNA was isolated from THP-1 cells, M0 macrophages, and M0 macrophages cocultured with L02-SCR or L02-Sh using TRIzol according to the manufacturer’s instructions. One microgram of RNA was reverse transcribed into cDNA using a First Strand cDNA Synthesis Kit. cDNA was quantified using the Applied Biosystems Step-One Real-Time PCR system with a SYBR Green Real-time PCR Master Mix kit. The following primer sequences that were used are shown in Table 1 (It is showed in Supplementary information). GAPDH served as the housekeeping gene.

**Table 1:** List of the sequence of gene primers.

Gene name	Forward (5′–3′)	Reverse (5′–3′)
IL-1β (mice)	CCAGGATGAGGACATGAGCA	CGGAGCCTGTAGTGCAGTTG
IL-4 (mice)	CGGCACAGAGCTATTGATGG	TCCGTGGATATGGCTCCTG
IL-6 (mice)	AGTTGCCTTCTTGGGACTGA	CCTCCGACTTGTGAAGTGGT
IL-10 (mice)	TAGAGCTGCGGACTGCCTTC	TTCCGATAAGGCTTGGCAAC
IL-12α (mice)	CTGGCGTCTACACTGCTGCT	CGTGATTGACACATGCTGGA
IL-12β (mice)	GCTGGTGTCTCCACTCATGG	TCTTCAGGCGTGTCACAGGT
TNF-α (mice)	TATGGCTCAGGGTCCAACTC	CCCATTTGAGTCCTTGATGG
CSF-1 (mice)	CGACTTCCCGTAAAGGCATA	AGCAGAGGGCACTTAAGCAA
TGF-α (mice)	CATTGATCTGCCCAGGTCTT	ACTTCTGCCTGGAGCTGTGT
TGF-β (mice)	TGCCCTCTACAACCAACACA	GTTGGACAACTGCTCCACCT
IFN-γ (mice)	CCTTTGGACCCTCTGACTTG	AAACAGCCATGAGGAAGAGC
HGF (mice)	CTCCCGAGAACTTCAAATGC	GCAGTAGCCAACTCGGATGT
VEGF (mice)	CCCTTCGTCCTCTCCTTACC	AAGCCACTCACACACACAGC
FPN1 (mice)	GCA GGC TCT GTT CTG GTC CT	GAT GAT TCC GCA GAG GAT GA
IL-1β (human)	ACGATGCACCTGTACGATCA	TCTTTCAACACGCAGGACAG
IL-4 (human)	GCCTTCAGCACATCTTCACACCTC	ATCGCTTCTCTGCACCTGTTCTTG
IL-6 (human)	GGTGTTGCCTGCTGCCTTCC	GTTCTGAAGAGGTGAGTGGCTGTC
IL-10 (human)	TGCCTTCAGCAGAGTGAAGA	GTCTTGGTTCTCAGCTTGGG
IL-12α (human)	GAGTTCAAGACCAGCCTGACCAAC	ACCTCCACCTCCGAGTTCAAGC
IL-12β (human)	GAGCAGGCATCAGCACCATCTG	ACACCATCAGCAGCATCACCTTG
TNF-α (human)	AGCTGGTGGTGCCATCAGAGG	TGGTAGGAGACGGCGATGCG
CSF-1 (human)	CAGAAGGAGGACCAGCAAGTGAAG	GCCAGCAAGACCAGGATGACAC
TGF-α (human)	GCCTCTGCCGATCTTGAACATCTC	TGCCTACACCTACCTGCTTACCTG
TGF-β (human)	CACGTGGAGCTGTACCAGAA	GAACCCGTTGATGTCCACTT
IFN-γ (human)	TGTTACTGCCAGGACCCATA	CTTCCTTGATGGTCTCCACA
HGF (human)	AATGGCACGATCTTGGCTCACTG	AGGAGTGGTGGTGGCAGGTG
VEGF (human)	TAGCTGCCTGCCTGGTGACTG	CAGAAGGACCACAGGACACAACAC
FPN1 (human)	TGA ATG CCA CAA TAC GAA GG	CCA AGT TCC ATC CCG AAA TA
β-Actin	GCC ACT GCC GCA TCC TCT TC	AGC CTC AGG GCA TCG GAA CC
14146	GGC ATT CCC AAC ACT TTA GC	
14147	CCC ATA GGT TAA ACT GCT TCA A	
20239	TGCAAACATCACATGCACAC	
20240	TTGGCCCCTTACCATAACTG	
olMR5374	GAAGCAGAAGCTTAGGAAGATGG	

### ELISA

The levels of *IL-4, IL-10, TGF-β, TNF-α, and IFN-γ* (Cell signaling Company, USA) in the serum and cell culture supernatant were measured by ELISA following the manufacturer’s instructions. All ELISA reagent kits were purchased from ExCellBio. All samples were measured in triplicate. The concentration of certain cytokines in the serum and cell culture was quantified by standard curve. The fold change in the expression of cytokines was calculated compared to the control group.

### Agarose gel electrophoresis

Agarose Gel Electrophoresis was performed as described previously^38^. DNA was extracted using a GeneJET Genomic DNA Purification kit (Thermo, USA). Extracted DNA samples were amplified by PCR of the *FPN1* gene by restriction fragment length polymorphism-PCR. The PCR mixture contained 12 µL of OneTaq Quick-Load 2X Master Mix with standard buffer, 4 µL of each primer (40 µM), and 8 µL of DNA sample (< 10 ng), resulting in a total volume of 28 µL. The reaction was carried out under the following conditions: initial denaturation at 95 °C for 15 min followed by 34 cycles of denaturation at 95 °C for 1 min, annealing at 56 °C for 1 min and elongation at 72 °C for 1 min with a final extension at 72 °C for 10 min. Gel electrophoresis was performed on 1.5 ~ 2% agarose gels supplemented with 11 mM MgCl_2_ and SYBER™ safe DNA gel stain in TAE buffer (0.5×) at pH 8. The samples of interest were mixed with 20% loading buffer (6×) and then loaded into agarose gel wells. The gel was run at 70 V for 2.5–3 h and visualized using a UVP scanner.

### Western blot

The cells were washed with ice-cold PBS and then lysed in ice-cold RIPA lysis buffer containing 1 mmol/L PMSF. The protein concentrations were calculated using BCA assay kits. A total of 40 μg of total cellular protein was subjected to 8% SDS-PAGE and transferred to a PVDF membrane. The membrane was blocked with 5% defatted milk powder at room temperature for 1.5 h, incubated with primary antibodies (*FPN1*, *CD11b*, *CD206*, *iNOS*, and *GAPDH* were come from Cell Signaling Company, USA) at 4 °C overnight and then incubated with HRP-conjugated secondary antibodies at room temperature for 1.5 h. Following each step, the membranes were washed five times with TBS-T for 5 min. Finally, the blots were developed using the enhanced chemiluminescence system.

### Immunohistochemical analysis

Immunohistochemical analysis was played according to the procedure reported by Hau-Wen Li^39^*.* Deparaffinization of the tissue sections was executed using a graded ethanol series, and then 0.3% hydrogen peroxide was used to block endogenous peroxidase activity. Antigen retrieval was achieved by placing the sections in 10 mmol/L citrate buffer (pH 6.0) and heating in an autoclave for 5 min. The sections were then rinsed with phosphate-buffered saline (PBS, pH 7.2) and incubated with 10% nonimmunized goat serum for 30 min at room temperature to inhibit nonspecific binding. The tissue sections were then incubated at 4 °C overnight with antibodies. After 24 h, the slides were incubated with horseradish peroxidase (HRP)-conjugated anti-mouse/rabbit secondary antibodies for 30 min at 25 °C. Then, staining with 3,3-diaminobenzidine (DAB) was carried out for 90 s at room temperature; hematoxylin staining was performed as a control.

### Histology

Tissue from mice was fixed with 4% paraformaldehyde, embedded in paraffin, and sectioned (thickness = 5 μm). Masson staining was conducted by a Masson Stain Kit (Solarbio, Beijing, China). Sirius red staining was performed to visualize collagen fibers using a Sirius Red Stain Kit (Solarbio, Beijing, China). Each section was assessed under light microscopic fields.

### Non-parenchymal cells isolation

Five to six mice livers in each experiment were excised, finely smashed by forceps in Gey’s balanced salt solution (GBSS), placed in a flask containing 0.16 mg/mL collagenase IV and 10 μg/mL DNase I in a shaking water bath at 37 °C for 10 min. The resulting cell suspension was sequentially passed through a lsyer of 150-mesh nylon gauze to remove undigested tissue, and 100-μm cell strainer. The resulting single-cell suspension was centrifuged at 50 × *g* for 3 min several times to eliminate hepatocytes. The supernatant, enriched in hepatic stellate cells, Kupffer cells and liver sinusoidal endothelial cells, was then centrifuged for 10 min at 400 × *g*. The pellet was washed twice with PBS and resuspended for further antibody incubation^40^.

### Flow cytometry

*F4/80*^+^*CD16/32*^+^*,* and *F4/80*^+^*CD206*^+^ cells were quantified by antibody labeling and flow cytometry. Freshly isolated liver cells were resuspended in 500 μL PBS with 0.25% EDTA-trypsin, incubation with 5 μL of FITC conjugated anti-F4/80, APC-conjugated anti-CD16/32, and PE-conjugated anti-CD206 antibodies at room temperature for 30 min and resuspended 2–3 times during incubation. Anti-mouse FcR mAb was used prior to staining to block any non-specific FcR binding. Afterward, cells were washed twice with PBS and resupended in 150 μL PBS for analysis. The data were acquired on an LSRII and analyzed by FlowJo v8.8.6.

### Antibodies and reagents

The antibody to FPN1 (#L3266) was obtained from Santa Cruz Biotechnology (USA). The antibody to GAPDH (#5174) was obtained from cell signaling Company (USA). The antibody to Collagen-1 (#ab34710), Collagen-4 (#ab6586), Laminin (#ab11575), MMP9 (#ab38898), a-SMA (#ab5694), VEGF (#ab46154), TGF-B (#ab92486), INOS (#ab15323) were obtained from abcam (USA). The antibody to F4/80 (#123107) and CD206 (#141706) were obtained from Biolegend. The antibody to F4/80 (#12-4801-82), CD68 (#11-0689-42) and CD16/32 (#4303632) were obtained from Invitrogen. Sirus Red Staining Kit (#G1471) was obtained from Solarbio (China) and Masson Staining Kit (#D026-1-3) was obtained from NanJing JianCheng (China).

### Iron assay

The relative iron concentration in cell lysates was assessed using an Iron Assay Kit (#ab83366, Abcam) according to the manufacturer’s instructions.

### Statistical analysis

Statistical analysis was performed using GraphPad Prism software version 6.0. All data are expressed as the mean ± standard deviation (SD). Comparisons between two groups were performed using Student’s *t* test, and three or more groups were evaluated for significance using one-way ANOVA combined with Bonferroni's post hoc test. A *P* value < 0.05 or 0.001 considered statistically significant.

### Ethical approval

The housing of the animals and the experiment procedures were carried out in accordance with the Guide for the Care and Use of Laboratory Animals (United States National Institutes of Health) and were approved by the Ethical Committee for Care and Use of Laboratory Animals of Guangzhou Medical University. This article does not contain any studies with human participants performed by any of the authors.

## Supplementary Information

Supplementary Information.

## Data Availability

The datasets used in the current study are available from the corresponding authors on reasonable request.

## Abbreviations

IL-1β: Interleukin 1β
IL-4: Interleukin 4
IL-6: Interleukin 6
IL-10: Interleukin 10
IL-12α: Interleukin 12α
IL-12β: Interleukin-12β
TNF-α: Tumor necrosis factor-α
CSF-1: Macrophage colony-stimulating factor
TGF-α: Transforming growth factor-α
TGF-β: Transforming growth factor-β
IFN-γ: Interferon γ
HGF: Hepatocyte growth factor
VEGF: Vascular endothelial growth factor
FPN1: Ferroportin
