# Supplementary material for: Decreased ferroportin in hepatocytes promotes macrophages polarize towards an M2-like phenotype and liver fibrosis
Source: Sci Rep. 2021 Jun 28;11:13386. doi: 10.1038/s41598-021-92839-z (PMC8239022; doi:10.1038/s41598-021-92839-z)
Supplement: Supplementary file 1 — Supplementary Information. [file 41598_2021_92839_MOESM1_ESM.docx]

**Decreased ferroportin in hepatocytes promotes macrophages polarize towards an M2-like phenotype and liver fibrosis**

**Chengyuan Cai*,**

Key Laboratory of Molecular Target & Clinical Pharmacology and the State Key Laboratory of Respiratory Disease,  School of Pharmaceutical Sciences & The Fifth Affiliated Hospital , Guangzhou Medical University, Guangzhou, 511436, PR China

**Danning Zeng*,**

The Second Affiliated Hospital, Guangzhou Medical University, Guangzhou, Guangdong, China 510260

**Qing Gao,**

Department of Healthy Food Development, Infinitus (China) Company Ltd., Guangzhou, Guangdong 510024;

**Lei Ma**

Key Laboratory of Molecular Clinical Pharmacology & Fifth Affiliated Hospital, Guangzhou Medical University, Guangzhou 511436, Guangdong, China

**Bohang Zeng**

The Second Affiliated Hospital, Guangzhou Medical University, Guangzhou, Guangdong, China 510260

**Yi Zhou^#^,**

Key Laboratory of Molecular Target & Clinical Pharmacology and the State Key Laboratory of Respiratory Disease,  School of Pharmaceutical Sciences & The Fifth Affiliated Hospital , Guangzhou Medical University, Guangzhou, 511436, PR China

**He Wang^#^**

The Second Affiliated Hospital, Guangzhou Medical University, Guangzhou, Guangdong, China 510260

*Chengyuan Cai and Danning Zeng contributed equally to this work

**^#^**Corresponding author:

**He Wang**, The Second Affiliated Hospital, Guangzhou Medical University, Guangzhou, Guangdong, China, Phone: 86-20-34153146; Fax: 86-20-34153146; E-mail: wanghe97@gzhmu.edu.cn;

**Yi Zhou**, Key Laboratory of Molecular Target & Clinical Pharmacology and the State Key Laboratory of Respiratory Disease,  School of Pharmaceutical Sciences & The Fifth Affiliated Hospital , Guangzhou Medical University, Guangzhou, 511436, PR China, Phone: 86-20-37103268; Fax: 86-20-37103268; Email: zhouyi0264@gzhmu.edu.cn

Conflict of interest: The authors declare no conﬂict of interest.

Running title: Antiliver Fibrosis Effects of Ferroportin

**1. Materials and methods**

**1.1 Real time Quantitative PCR**

Total RNA was isolated from THP-1, M0, and two kinds of M0 macrophages co-cultured with L02-SCR or L02-sh using TRIzol, according to the manufacturer’s instructions. 1 ug RNA was reverse transcribed into cDNA using a First Strand cDNA Synthesis Kit. cDNA was quantified using Applied Biosystems Step-one Real-Time PCR system with a SYBR Green real-time PCR Master Mix kit. The following primer sequences were used as shown in Table 1. GAPDH served as the housekeeping gene. For example, figure 1-8. N.S indicates no significance.

**1.2 ELISA**

The serum and cell culture supernatant level of *IL-4, IL-10, TGF-β, TNF-α, IFN-γ* were measured by ELISA following the manufacturer’s instructions. All the ELISA reagent kits were purchased from ExCellBio. All samples were measured in triplicate. The concentration of certain cytokines in serum and cell culture was quantified by standard curve. The expression fold change of the cytokines was calculated compared to control group. N.S indicates no significance. For example, figure 9-11

**Figures:**


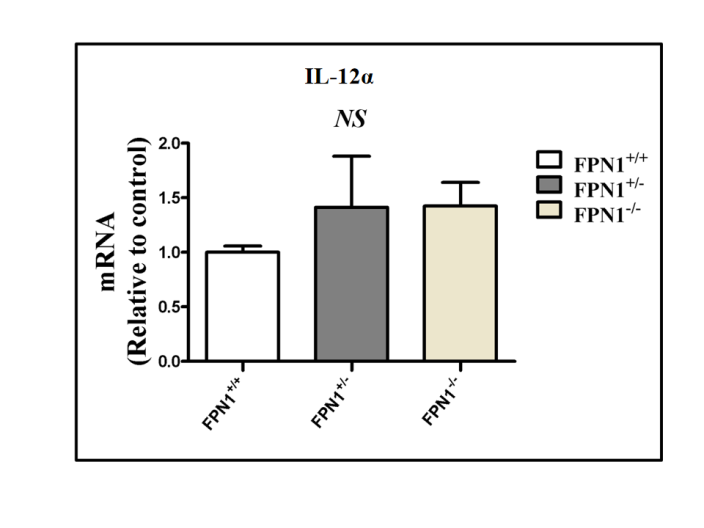


Figure S1


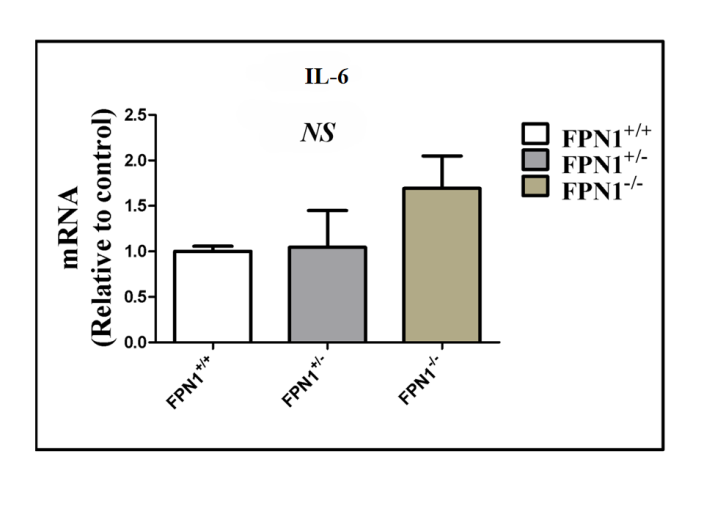


Figure S2


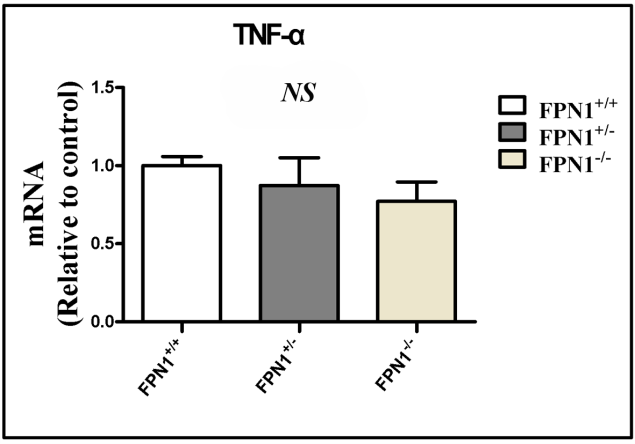


Figure S3


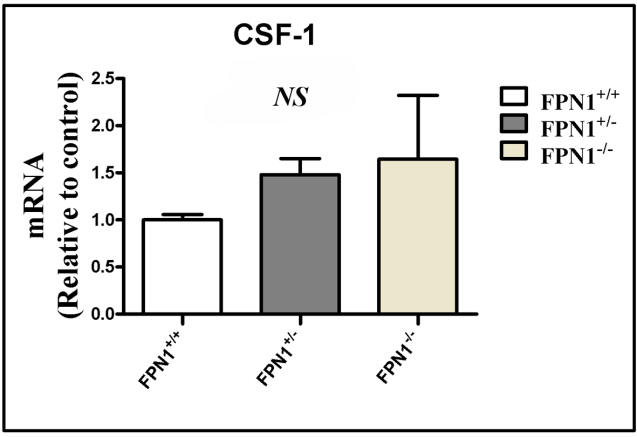


Figure S4


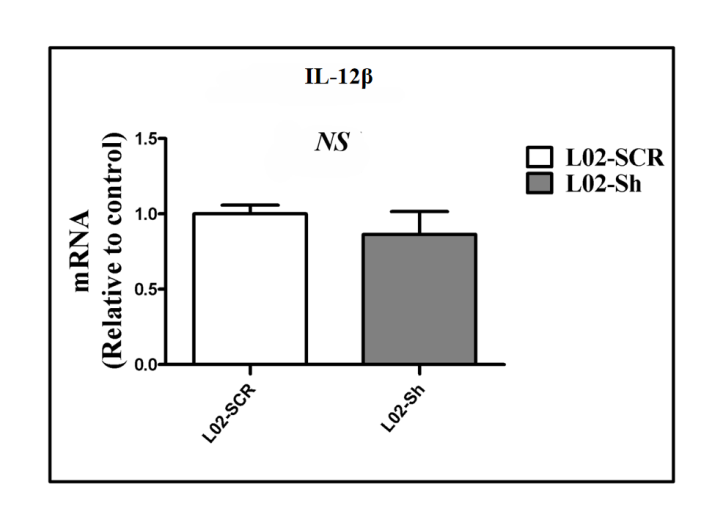


Figure S5

Figure S1-5: Q-PCR analysis of the expression of specific biomarkers (Figure S1, S2, S3, S4, S5) in mouse livers. *NS* indicated no significance.


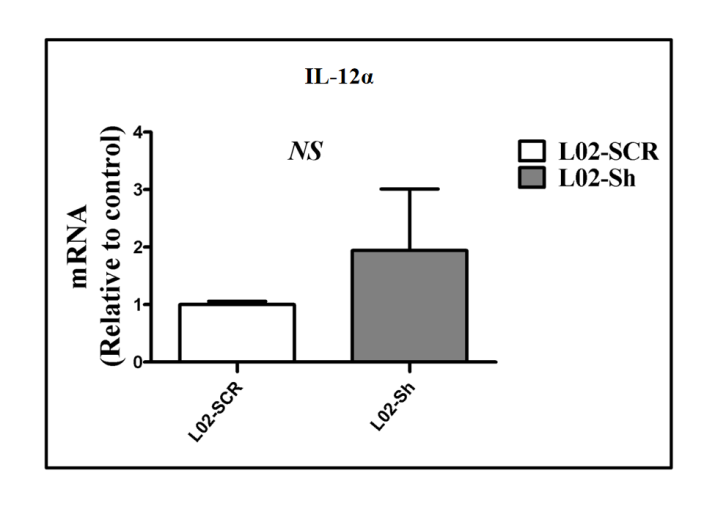


Figure S6


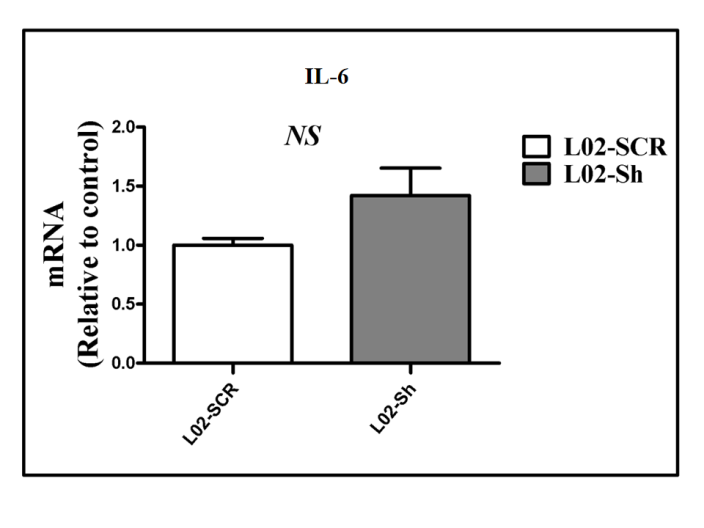


Figure S7


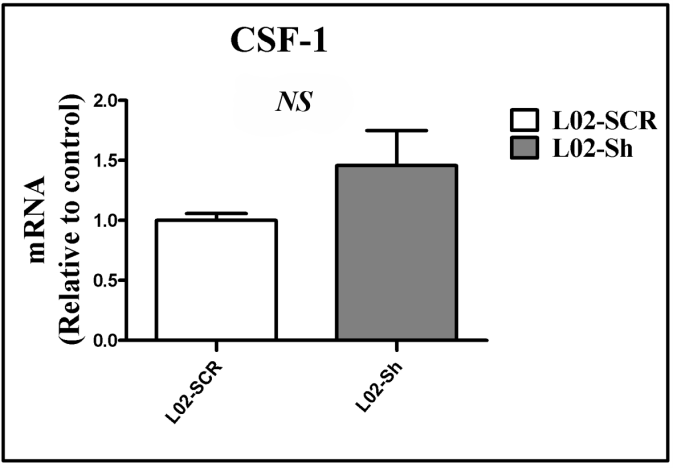


Figure S8

Figure S6-8: Q-PCR analysis of the expression of specific biomarkers (Figure S6, S7, S8) in L02-SCR cells and L02-Sh cells. *NS* indicated no significance.


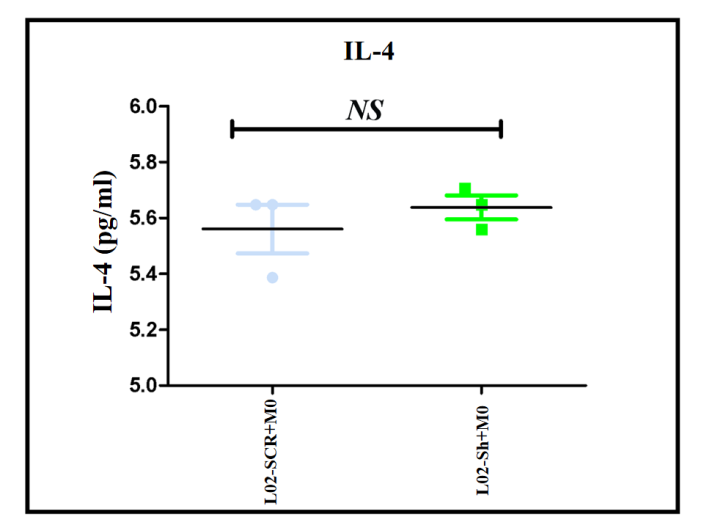


Figure S9


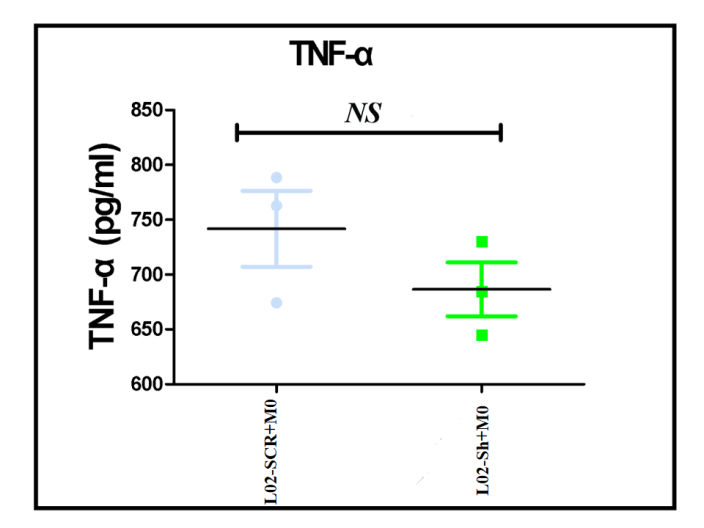


Figure S10


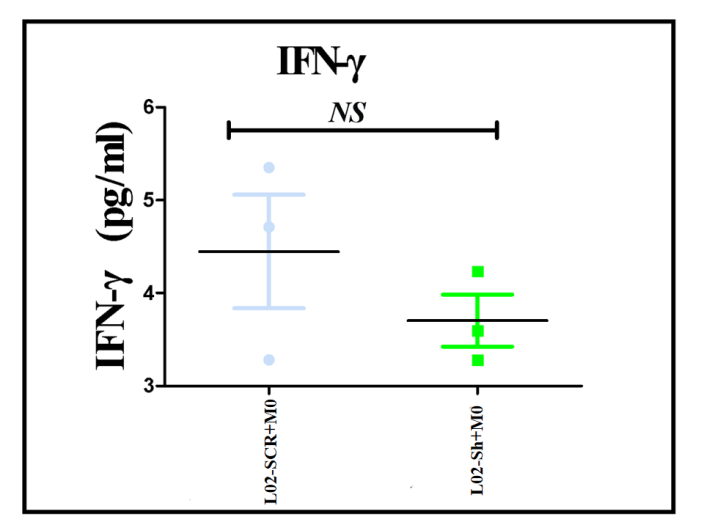


Figure S11

Figure S9-11: ELISA analysis of the expression of specific biomarkers (Figure S9, S10, S11) in the culture medium. *NS* indicated no significance.


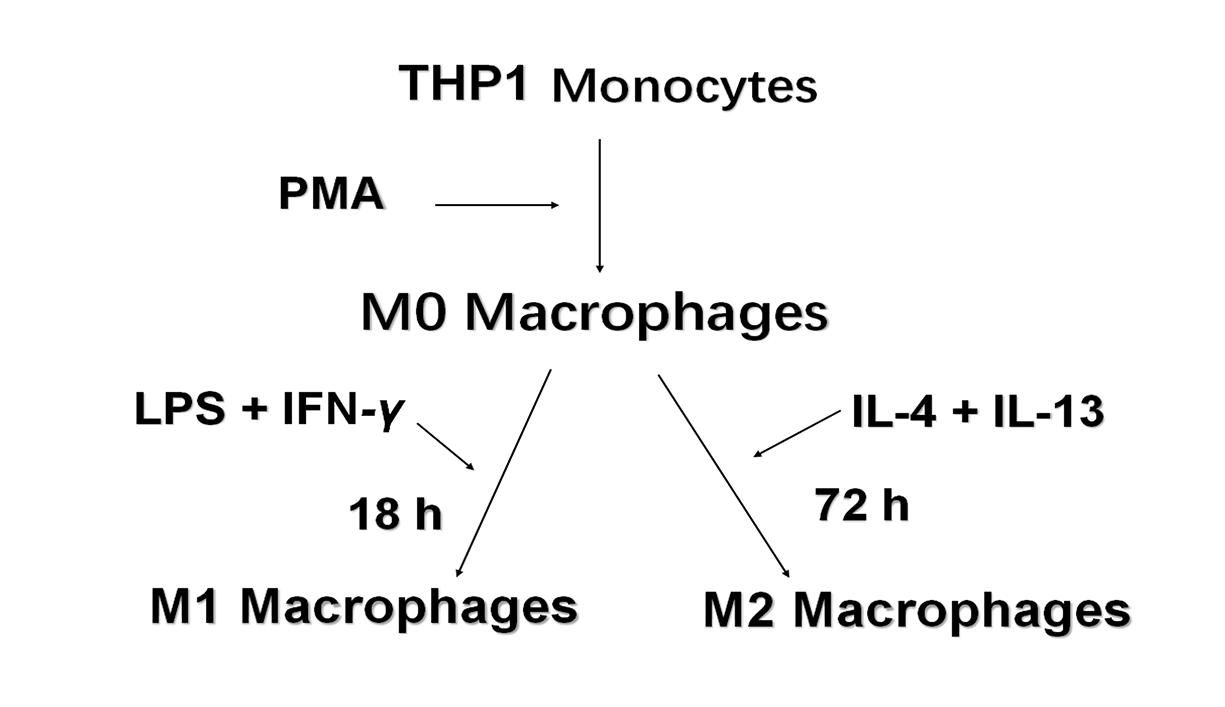


Figure S12

PMA induced suspended THP1 cells to M0 macrophages, which wound transfer to M1 or M2 under different condition.


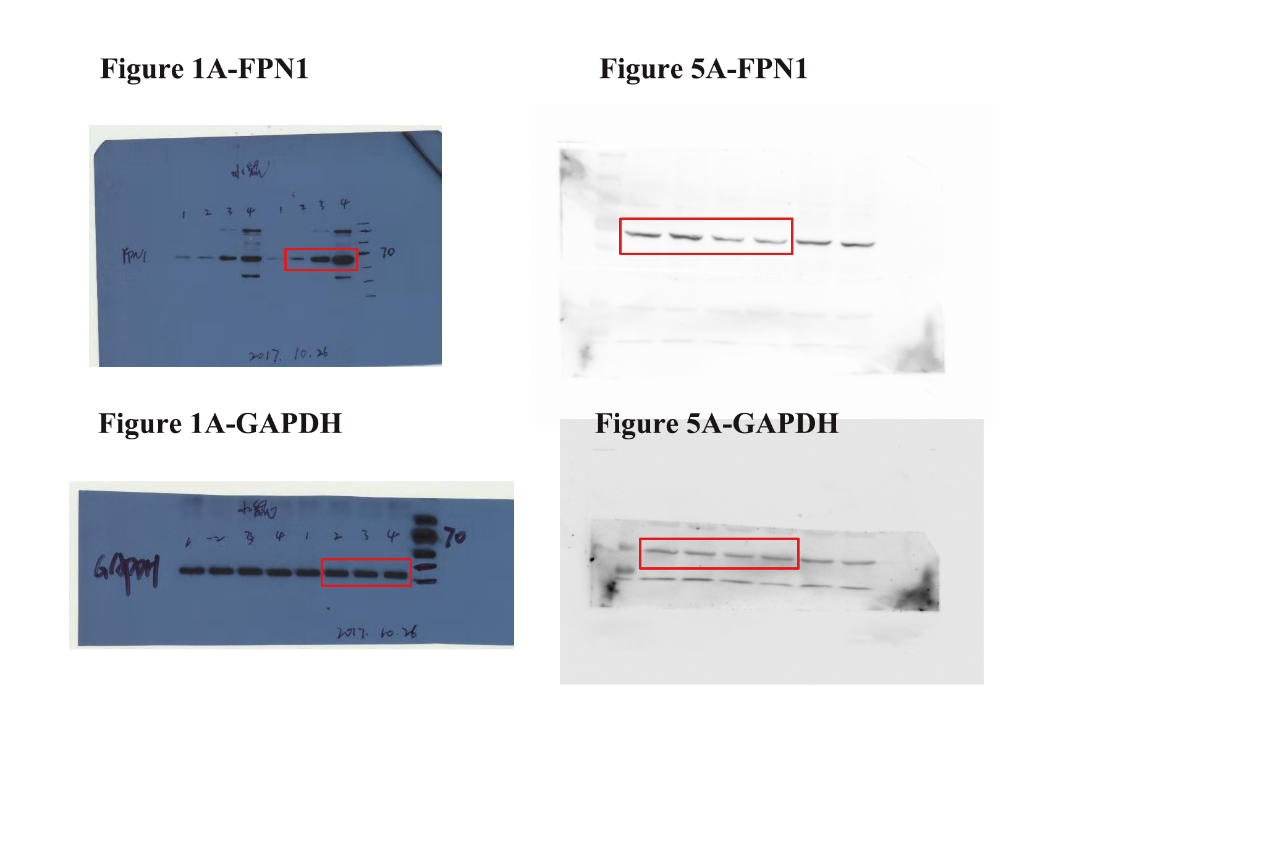

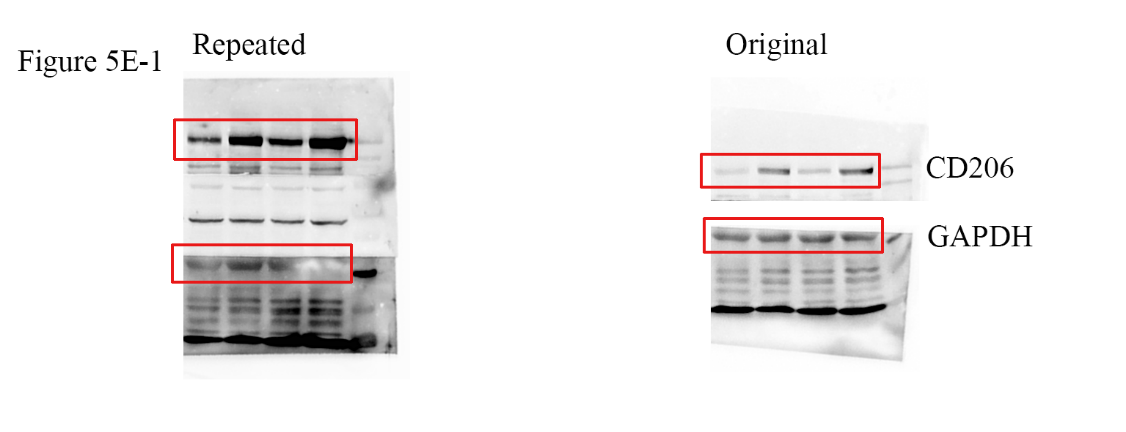


Original materials
